# A dual-marker peripheral signature of IL-6 elevation and *NEAT1* reduction in negative-symptom schizophrenia: a cross-sectional study

**DOI:** 10.1017/neu.2026.10055

**Published:** 2026-01-23

**Authors:** Cosmin Ioan Moga, Paul Adrian Chiroi, Livia Budișan, Octavia Oana Căpățînă, Cătălina Angela Crișan, Mihaela Fadgyas-Stănculete, Ioana Berindan-Neagoe, Ioana Valentina Micluția

**Affiliations:** 1 Neurosciences, Iuliu Hațieganu University of Medicine and Pharmacy, Romania; 2 Genomics Department of MedFuture – Institute of Biomedical Research, Cluj-Napoca, Romania, Iuliu Hațieganu University of Medicine and Pharmacy, Romania

**Keywords:** Negative symptoms, schizophrenia, inflammation, IL-6, NEAT1

## Abstract

**Background:**

Schizophrenia (SCZ) shows marked biological heterogeneity, with negative symptoms linked to poor outcomes and hypothesised immune dysregulation. This study examined whether a peripheral cytokine–long non-coding RNA (lncRNA) panel could distinguish patients with SCZ and Brief Negative Symptom Scale (BNSS)-defined subgroups from healthy controls (HC).

**Methods:**

Forty-one hospitalised patients with SCZ completed the BNSS and the Positive and Negative Syndrome Scale (PANSS). Twenty HCs, frequency-matched for age and sex, served as comparison samples. Severe negative-symptom subgroups were defined using two BNSS criteria: a broader (SNS1) and a more restrictive (SNS2) threshold. Serum cytokines – interleukin-6 (IL-6), tumour necrosis factor-α (TNF-α), interleukin-10 (IL-10) – and leukocyte lncRNAs (*MALAT1*, *NEAT1*, *MEG3*) were quantified by enzyme-linked immunosorbent assay and quantitative RT-PCR. Covariate-adjusted logistic and multinomial models (adjusting for age, sex, body mass index, and smoking) assessed discrimination using area under the receiver-operating-characteristic curve (AUC) and interquartile-range odds ratios (OR_IQR).

**Results:**

IL-6 correlated with PANSS Total (*ρ* = 0.48, *p* = 0.001) and Negative (*ρ* = 0.34, *p* = 0.032) scores and was higher in SCZ than HC (*p* = 0.033), with further increases in SNS subgroups. *NEAT1* was significantly reduced only within BNSS-defined subgroups (*p* ≤ 0.025). The dual-marker pattern (IL-6 ↑, *NEAT1* ↓) showed the strongest discrimination for SNS1 versus HC (AUC = 0.85) and the steepest multinomial contrasts for SNS2 (IL-6 OR_IQR = 4.98; *NEAT1* OR_IQR = 0.11).

**Conclusions:**

Elevated IL-6 and decreased *NEAT1* define a peripheral signature linked to negative-symptom severity in SCZ and may support biologically informed stratification and longitudinal research.

Significant outcomes

- Among cytokines, IL-6 was the only marker showing consistent associations with diagnosis and symptom burden, with higher levels in SCZ and the BNSS-defined severe negative-symptom subgroups.
- *NEAT1* downregulation, observed only within BNSS-defined negative-symptom subgroups compared to HC, provided additional discriminative value beyond cytokines and showed a graded decrease with increasing severity, whereas *MALAT1* and *MEG3* showed no association with negative symptoms or diagnosis, displaying only minor correlations with demographic variables.
- Discrimination versus HC increased with BNSS-defined negative-symptom severity. The dual-marker model combining IL-6 and *NEAT1* showed the highest accuracy in the broad BNSS subgroup (AUC = 0.85), while multinomial analyses revealed the most pronounced contrasts in the restrictive subgroup (IL-6 ↑, *NEAT1* ↓), following a graded continuum from SNS2 through non-SNS to HC.

Limitations

- The SCZ sample was modest and treatment-heterogeneous, composed entirely of hospitalised, chronically treated patients, while the HC group was small and selectively recruited, limiting generalisability to broader and community-based populations.
- The cross-sectional design limits causal interpretation between symptoms and biomarkers, and whole-blood profiling lacked cellular and isoform specificity, underscoring the need for pathway-level and single-cell validation.
- We operationalised negative symptoms using BNSS-derived definitions (SNS1/SNS2) instead of longitudinal constructs like persistent negative symptoms or deficit SCZ, usually assessed with the Schedule for the Deficit Syndrome (SDS). This choice aimed for a practical inpatient screening tool but may affect comparability with SDS-based literature.

## Introduction

Schizophrenia (SCZ) is a heterogeneous disorder encompassing multiple clinical phenotypes, with negative symptoms being most strongly associated with a poor prognosis and the traditionally debilitating ‘Kraepelinian’ course (Kraepelin, 1921; Galderisi *et al*., 2018). Contemporary frameworks parse negative symptoms into five domains **–** anhedonia, asociality, avolition, blunted affect, and alogia – that load onto a two-factor structure comprising motivation–pleasure (MAP) and expressive deficits (EXP) (Kirkpatrick *et al*., 2006). Beyond the behavioural and cognitive phenotypes, convergent genomics, imaging, and fluid-biomarker work implicate a peripheral–central nervous system (CNS) immunoinflammatory axis in SCZ, with alterations in cytokine profiles detected in blood and brain (Goldsmith *et al*., 2016; Hudson & Miller, 2018; Pandey *et al*., 2018; Lanz *et al*., 2019; Dawidowski *et al*., 2021). In parallel, long noncoding RNAs (lncRNAs) have emerged as regulators of immune and neural processes and are increasingly implicated in neuropsychiatric disease, including SCZ (Mishra & Kumar, 2021; Cao *et al*., 2023; Mousavinejad *et al*., 2025).

Cytokines are pleiotropic immune proteins produced by immune and non-immune cells that act peripherally and within the CNS (Frydecka *et al*., 2018). Pro-inflammatory mediators such as interleukin-6 (IL-6) and tumour necrosis factor-α (TNF-α), and the anti-inflammatory interleukin-10 (IL-10) are central to this axis. IL-6, signalling via gp130-containing receptor complexes (Rose-John, 2018), is frequently elevated in SCZ and is often interpreted as a state marker of inflammatory load (Reale *et al*., 2021; Zhou *et al*., 2021). TNF-α, which engages TNFR1/TNFR2 and modulates immune defence (Idriss & Naismith, 2000), is also commonly increased in SCZ (Goldsmith & Rapaport, 2020; Maes *et al*., 2020), though treated individuals can show attenuation (Lv *et al*., 2015). IL-10, functioning via JAK/STAT-mediated negative feedback mechanisms, constrains NF-κB–driven transcription (Moore *et al*., 2001); results concerning IL-10 in SCZ are inconsistent, demonstrating increases, decreases, or null effects across different studies (Gao *et al*., 2014; Xiu *et al*., 2014; Malashenkova *et al*., 2021). Together, IL-6, TNF-α, and IL-10 provide a focused framework for interrogating immune imbalance in SCZ.

LncRNAs are transcripts longer than 200 nucleotides with low protein-coding potential that regulate gene expression through epigenetic mechanisms, transcriptional scaffolding, and post-transcriptional control (Liao *et al*., 2018). In peripheral blood, lncRNAs influence both innate and adaptive immune signalling, thereby affecting cytokine production (Plewka & Raczynska, 2022; Zeng *et al*., 2023). We focused on three nuclear-enriched lncRNAs with complementary roles in nuclear RNA organisation and documented immunomodulatory links relevant to neuroinflammation and SCZ. *MALAT1* (metastasis-associated lung adenocarcinoma transcript 1; also known as *NEAT2*) is a conserved nuclear-speckle lncRNA that coordinates transcription–splicing coupling (Galganski *et al*., 2017). In immune models, *MALAT1* generally acts as a regulatory or anti-inflammatory brake, as its loss can de-repress NF-κB targets and increase IL-6 expression (Plewka & Raczynska, 2022; Cao *et al*., 2023). In the context of SCZ-related research, Li et al. reported decreased *Malat1* expression in the prefrontal cortex of MK801- and methamphetamine-induced mouse models and lower peripheral blood *MALAT1* in untreated patients with SCZ, with partial normalisation following antipsychotic treatment (Li *et al*., 2018). *NEAT1* (nuclear enriched abundant transcript 1) produces two isoforms: a short *NEAT1*_*1* and a long *NEAT1*_*2*, with the latter essential for paraspeckle assembly (Yamazaki & Hirose, 2015; Almalki, 2024). Paraspeckles are nuclear bodies, alongside the speckles, that buffer transcriptional stress by sequestering RNA-binding proteins and retaining specific RNAs (Yamazaki & Hirose, 2015). *NEAT1* has been associated, in a context-dependent manner, with pro-inflammatory signalling and changes in the blood–brain barrier (BBB) (Zhang *et al*., 2019, 2025; Almalki, 2024). Cortical *NEAT1* downregulation has been reported in postmortem brain tissue from patients with SCZ (Katsel *et al*., 2019). In the same study, *Neat1* deficiency in mice was associated with reductions in oligodendrocyte-lineage cells in the frontal cortex and altered pathways involved in oligodendrocyte differentiation and myelination; co-expression with SOX10 further supported this association (Katsel *et al*., 2019). Consistent with these findings, another study observed that peripheral *NEAT1* was decreased in untreated patients with SCZ and approached control levels after treatment (Li *et al*., 2018). Collectively, these data suggest *NEAT1* involvement at the neuroimmune–myelin interface, although mechanistic pathways remain to be elucidated. *MEG3* (maternally expressed gene 3) relocates to nuclear speckles under transcriptional stress (Hasenson *et al*., 2022), such as in inflammatory environments. Immunologically, it can exert pro- or anti-inflammatory effects depending on cell type and stimulus (Zeng *et al*., 2023). Altered *MEG3* expression has been observed in SCZ; a case–control study reported increased peripheral expression in patients, significant only in females, suggesting possible sex-specific regulation (Fallah *et al*., 2019). Moreover, in a cohort of patients diagnosed with psychosis, peripheral *MEG3*, along with *PINT* and *GAS5*, varied according to diagnostic status, symptom acuity, and treatment exposure. Notably, *MEG3* expression differed between drug-naïve and risperidone-treated patients, and risperidone modulated *MEG3* expression in vitro in M2^tol^ macrophages. (Sudhalkar *et al*., 2018).

Taken together, *MALAT1*, *NEAT1*, and *MEG3* form a biologically relevant set for exploring a peripheral cytokine–lncRNA axis related to neuroimmune hypotheses in SCZ. Immune dysregulation in SCZ appears to vary across subgroups (Boerrigter *et al*., 2017; Zhu *et al*., 2022) and tends to be more pronounced in patients with prominent negative symptoms (Goldsmith *et al*., 2018; Dunleavy *et al*., 2022; Wang *et al*., 2022; Cyran *et al*., 2023). To identify relevant severe negative symptom groups, derivative constructs (e.g., persistent or predominant negative symptoms; deficit SCZ) have been proposed (Kirkpatrick *et al*., 2001; Buchanan, 2007), but they are complicated or require longitudinal confirmation. Therefore, we used the Brief Negative Symptom Scale (BNSS), developed according to the current five-domain, two-factor framework and practical for inpatient settings (Kirkpatrick *et al*., 2011), to phenotype hospitalised patients with SCZ while profiling a peripheral cytokine–lncRNA axis.

In this cross-sectional study, we aimed to develop and evaluate discriminative models using a peripheral cytokine–lncRNA panel to distinguish SCZ and BNSS-defined negative-symptom subgroups from healthy controls (HC). Specifically, we: (1) measured peripheral IL-6, TNF-α, and IL-10 as inflammatory predictors; (2) quantified the nuclear lncRNAs *MALAT1*, *NEAT1*, and *MEG3* to assess their incremental discriminative value beyond cytokines and their associations with BNSS severity; and (3) applied BNSS cutoffs to classify negative-symptom severity and tested whether biomarker-based discrimination versus HC increased with severity.

## Material and methods

### Participants

Patients diagnosed with SCZ were recruited from the open wards of the acute and chronic units of the Psychiatry Clinics I and II at Cluj-Napoca University Hospital. HCs were recruited by convenience sampling from hospital staff across different occupational categories and community volunteers. To approximate the demographic profile of the patient cohort, the HC group was frequency matched to patients by age (within 10-year bands) and sex, without individual one-to-one matching. All participants were personally approached and invited to participate by the study investigators.

Inclusion criteria for patients included (1) a DSM-5 diagnosis of SCZ confirmed through a structured clinical interview, (2) an age range of 18 to 65 years, and (3) clinical stability adequate to complete standardised psychometric assessments. Exclusion criteria encompassed: (1) presence of acute infections or recent traumatic injuries; (2) a history of autoimmune or chronic infectious diseases; (3) C-reactive protein levels 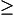 10 mg/l; (4) hematological abnormalities, including neutropenia associated with clozapine treatment (neutrophil count < 1500/mm^3^); (5) documented SARS-CoV-2 infection within the preceding 12 months, as elevated pro-inflammatory cytokines – particularly IL-6, IL-1β, and TNF-α – have been reported to persist for up to ten months post-infection and may reflect post-acute immune activation rather than illness-specific processes (Schultheiß *et al*., 2022); (6) intellectual disability (IQ < 70); and (7) comorbid psychiatric disorders or current substance use disorders. For controls, the exclusion criteria were identical to those for patients, with the additional requirement of no family history of severe psychiatric disorders (SCZ spectrum disorders and bipolar disorders) to minimise latent familial liability that may influence inflammatory and lncRNA measures.

All participants provided written informed consent. The study was approved by both the university and hospital ethics committees and conducted under the ethical principles outlined in the Declaration of Helsinki.

Socio-demographic data were collected from both the SCZ patients and the HC, including living environment, education level, marital status, and social (employment) status. Participants with SCZ provided additional information on the duration of illness and the daily dose of antipsychotics, converted into chlorpromazine (CPZ) equivalents. Additionally, body mass index (BMI) and smoking status – measured in cigarettes per day – were recorded as known factors affecting inflammation. All these socio-demographic variables and potential confounders were used as covariates in the subsequent analyses.

### Psychiatric measurements

Psychiatric symptoms were assessed using two validated clinician-rated instruments: the BNSS and the Positive and Negative Syndrome Scale (PANSS). The BNSS is a 13-item instrument designed to evaluate the five core domains of negative symptoms, along with the lack of normal distress. Each item is rated on a 7-point Likert scale (0–6), yielding a total score that can range from 0 to 78. The Romanian version was used with permission from the original authors, B. Kirkpatrick and G. Strauss, and has been validated for Romanian-speaking individuals diagnosed with SCZ in a previous study. The PANSS comprises 30 items, categorised into the Positive, Negative (scoring 7–49), and General Psychopathology (scoring 16–112) subscales. In this study, PANSS served mainly to quantify positive and general psychopathology, enabling comparisons with negative symptoms.

### Definitions of severe negative symptom (SNS) subgroups

The clinically relevant SCZ subgroups characterised by severe negative symptoms (SNS) were identified based on two parallel BNSS-based criteria described in the literature, representing a hierarchical spectrum from broad to restrictive definitions. The broad subgroup (SNS1) included patients with at least three BNSS items scored ≥ 3 or at least two items scored ≥ 4 (Galderisi *et al*., 2021). The restrictive subgroup (SNS2) required all five BNSS core domains (excluding ‘lack of normal distress’) to be scored ≥ 3 (Mucci *et al*., 2019). Accordingly, SNS2 cases also met the SNS1 criteria, whereas non-SNS2 cases included all remaining SCZ participants, including some who met the broad SNS1 definition and those with minimal negative symptoms. Non-SNS1, by contrast, referred explicitly to SCZ participants who did not meet the SNS1 criteria and thus represented the group with the least severe negative symptoms in the sample.

### Laboratory procedures

#### Blood collection

Venous blood was drawn from all participants within a standardised morning window (9:00–10:00) to minimise diurnal variation. For each participant, one 6 ml serum-separator tube was collected for cytokine quantification using enzyme-linked immunosorbent assays (ELISAs), and two 3 mL EDTA-coated Vacutainer tubes were collected for leukocyte RNA isolation and quantitative reverse-transcription PCR (qRT-PCR) of lncRNAs. Samples were immediately transported to the Genomics Department of MedFuture–Institute of Biomedical Research within the Iuliu Hațieganu University of Medicine and Pharmacy for further processing and molecular analysis.

#### ELISA procedures

The serum used for the ELISA test was obtained by centrifugation. Sandwich ELISA kits from Elabscience Biotechnology Inc. (Houston, USA) were used for quantification of the proteins in the blood samples (serum) for human IL-6 (cat.no. E-EL-H6156), human TNF-α (cat.no. E-EL-H0109), and human IL-10 (cat.no. E-EL-H6154. A Biotek Synergy H1 Hybrid absorbance microplate reader was used to conduct ELISA testing. Absorbance values were plotted against standard curves generated for each kit, and concentrations (pg/ml) were calculated using a four-parameter logistic regression model.

#### RNA extraction and qRT-PCR

Total RNA was extracted from peripheral blood leukocytes, obtained following red blood cell lysis from whole human blood, using the TRIzol reagent (TriReagent, Sigma–Aldrich, St Louis, MO, USA) according to the manufacturer’s protocol. RNA concentration and purity were assessed using a NanoDrop 1000 spectrophotometer (Thermo Scientific, Waltham, MA, USA). Complementary DNA (cDNA) synthesis was performed with the High-Capacity cDNA Reverse Transcription Kit (Thermo Fisher Scientific, Waltham, MA, USA). Quantitative real-time PCR was conducted for the lncRNAs *MALAT1*, *NEAT1*, and *MEG3*, using SYBR® Select Master Mix (Applied Biosystems, Waltham, MA, USA), with *B2M* and *RPLP0* as housekeeping (reference) genes (HK). Primer sequences for all target and reference genes are listed in Table 1. Threshold cycle (Ct) values were obtained via fluorescence detection and normalised against the geometric mean of the two HK genes. Subsequently, two types of fold change (FC) were computed, tailored to the analytical context objective: a. FC ΔCt: Used for within-group association analyses (e.g., among controls or patients with SCZ). FC ΔCt was calculated using the 2^−ΔCt^ method, where ΔCt = Ct target – Ct geomean (HK), and b. FC ΔΔCt: Used for between-group comparisons (e.g., SCZ vs. HC). FC ΔΔCt was computed using the 2^−ΔΔCt^ method, where ΔΔCt = ΔCt sample – mean (ΔCt HC).

**Table 1.** Primer sequences for target genes, including housekeeping and long non-coding RNA (lncRNA) transcripts

Target gene / lncRNA	Primer sequences
*B2M*	F: 5’ CACCCCCACTGAAAAAGATGAG 3’
	R: 5’ CCTCCATGATGCTGCTTACATG 3’
*RPLP0*	F: 5’ CCC AAT TGT CCC CTT ACC TT 3’
	R: 5’ ACC CAG CTC TGG AGA AGT CA 3’
*MALAT1*	F: 5’ AACTGCAGAGAGTTTGAGTGGTTTT 3’
	R: 5’ TGTCCTTATAGGCTGGCCATT 3’
*NEAT1*	F: 5’GAGAAAAGTCCAAAAGGAGCAC 3’
	R: 5’ GGATGAGGCCTGGTCTTGT 3’
*MEG3*	F: 5’ GGGTCTCTCCTCAGGGATG 3’
	R: 5’ATGGAGAGGAGGTGGTCCTT 3’

### Data transformation and analysis

All biomarker values were log-transformed to improve normality and stabilise variance before statistical modelling. Specifically, cytokine concentrations were log_10_-transformed (log(cytokine)), while RNA values were log_2_-transformed by calculating −ΔCt or −ΔΔCt, consistent with fold-change scaling conventions (Grund & Sabin, 2010). All subsequent statistical analyses were conducted on the log-transformed data. To ensure clarity and consistency throughout the study, two presentation methods were used: in the main text and tables, back-transformed values (geometric means) were reported as they are expressed – pg/ml for cytokines and FC ΔΔCt for lncRNAs. However, for −ΔCt values, back-transformation was not applied, as they represent within-sample log_2_-normalised expression levels rather than group-relative fold changes. In contrast, figures displayed the log-transformed scale to preserve the analytical context and facilitate interpretation of model coefficients.

Statistical analyses were conducted in R (version 4.2). Normality of continuous variables was assessed with the Shapiro–Wilk test, and data exploration used Spearman correlations to examine associations among symptoms, covariates, and biomarkers. Group differences were evaluated using t-tests or Wilcoxon tests for two groups, ANOVA or Kruskal–Wallis tests for multiple groups, and χ^2^ tests for categorical variables. For biomarker group comparisons on the log scale, effects are reported as ratios – geometric mean ratios (GMRs) for cytokines and fold-changes (FCs) for lncRNAs – with 95% CIs computed on the log scale and back-transformed; for Wilcoxon tests, CIs reflect back-transformed Hodges–Lehmann median differences. To assess discriminative performance, binary logistic regression models (logit link) with linear terms for biomarkers and covariates were fitted for three HC contrasts (SCZ versus HC, SNS1 versus HC, SNS2 versus HC). Multivariable models included biomarker predictors that showed significant or suggestive bivariate preliminary associations and were adjusted for age, sex, BMI, and smoking status (coded yes/no for simplicity). Model assumptions were checked for multicollinearity (variance inflation factors), influence (Cook’s distance), and potential separation (coefficient magnitudes and standard errors). Discrimination was summarised by the area under the ROC curve (AUC, DeLong 95% CI), accuracy by the Brier score, and calibration by the slope and intercept. Wald and likelihood-ratio (LR) tests were reported for individual predictors, and interquartile-range odds ratios (OR_IQR = exp[β × IQR]) were calculated for continuous predictors, where IQR is the 75th–25th percentile on the analysis scale. As a complementary sensitivity analysis, multinomial logistic regression models were fitted across all participant groups (HC, non-SNS, and SNS) within a unified framework to examine whether biomarker effects were consistent and reflected a dimensional rather than categorical organisation. Figures were generated in R (packages: ggplot2, patchwork, ggraph) and finalised in Adobe Illustrator (version 29.6.1) for panel layout and sizing.

## Results

### Preliminary exploratory analyses

#### Sample characteristics

A total of 20 HC participants and 41 individuals with SCZ were included. Groups were comparable in age (HC: *M* = 40.70, *SD* = 10.12; SCZ: *M* = 42.10, *SD* = 11.60) and sex distribution (approximately 1:1). Compared with HC, patients had lower educational attainment and were more frequently single and unemployed (see Table 2).

**Table 2.** Demographic & clinical characteristics and biomarkers

Demographic & clinical characteristics	HC (*n* = 20)	SCZ (*n* = 41)	Difference	Biomarkers	HC (*n* = 20)	SCZ (*n* = 41)	Difference
Sex (Female)	10 (50.0%)	23 (56.1%)	*χ* ^2^ = 0.20, *p* = 0.654	Cytokines (pg/ml)			
Age (years)	40.70 ± 10.12	42.10 ± 11.60	*W* = 386.5, *p* = 0.723	IL-6	1.38 ± 1.45	1.70 ± 1.54	*W* = 271, *p* = 0.033
Living Environment (Urban)	18(90.0%)	33 (80.5%)	FET, *p* = 0.474	TNF-*α*	9.54 ± 2.15	6.42 ± 1.49	*W* = 504.5, *p* = 0.148
Education Level (Secondary)	6 (30.0%)	27 (65.9%)	*χ^2^* = 7.38, *p* = 0.026	IL-10	1.84 ± 1.31	1.90 ± 1.54	*W* = 349.5, *p* = 0.354
Marital Status (Single)	8 (40.0%)	29 (70.7%)	*χ^2^* = 5.31, *p* = 0.021	lncRNA (FC ΔΔCt)			
Social Status (Unemployed)	1 (5.0%)	36 (87.8%)	*χ^2^* = 5.31, *p* < 0.001	*MALAT1*	1.00 ± 1.90	1.16 ± 1.75	*W* = 345, *p* = 0.321
BMI (kg/m^2^)	28.26 ± 5.97	28.65 ± 8.20	*W* = 413, *p* = 0.969	*NEAT1*	1.00 ±2.28	0.65 ± 2.22	*t* = 1.91, *p* = 0.064
Smoking (cigarettes/day)	4.75 ± 7.86	9.05 ± 12.09	*W* = 330.5, *p* = 0.176	*MEG3*	1.00 ± 5.01	1.20 ± 3.18	*t* = −0.45, *p* = 0.655
Duration of illness (years)	—	16.83 ± 10.61					
Chlorpromazine equivalent doses (mg/day)	—	511.18 ± 265.31					

*Note:* HC, healthy control group; SCZ, full schizophrenia group. Values are presented as *n* (%) for categorical variables and mean ± *SD* for continuous variables. Biomarker means are geometric (back-transformed from log-transformed data); Test statistics and *p*-values for differences between the two groups of biomarkers were calculated using log-transformed data: *χ^2^* = chi-square test statistic (for categorical variables); FET, Fisher’s Exact Test (used for small expected cell counts); *t,* Welch’s t-test statistic (used for normally distributed variables, per Shapiro–Wilk test); *W,* Wilcoxon rank-sum statistic (used for non-normal distributions).

#### Negative symptom profiles

The mean total PANSS score was 93.44 (*SD* = 17.61), including a Negative subscale score of 27.27 (*SD* = 8.23). The mean BNSS total score was 40.29 (*SD* = 17.24). Based on the BNSS criteria, 27 individuals (65.9%) met the criteria for the SNS1 group, while 14 individuals (34.2%) met the more stringent SNS2 definition. The mean BNSS total score was 50.04 (*SD* = 10.70) in the SNS1 subgroup and 57.00 (*SD* = 6.67) in the SNS2 subgroup (see Table 3). Symptom–covariate analyses revealed that male participants exhibited significantly higher scores for lack of normal distress (median difference [male − female] = 1.00, 95% CI [0.00, 2.00], *p* = 0.039). Additionally, BMI was inversely correlated with most negative symptom dimensions, with *ρ* values ranging from –0.33 to –0.43 (*p* < 0.05). CPZ equivalent doses were also inversely correlated with anhedonia (*ρ* = –0.39, *p* = 0.011) and MAP score (*ρ* = –0.38, *p* = 0.015). Detailed results are shown in Supplementary Figure S1.

**Table 3.** Symptom scores across the full schizophrenia sample and the two severe negative symptom subgroups

Symptom	SCZ (*n* = 41)	SNS1 (*n* = 27)	SNS2 (*n* = 14)
PANSS total	93.44 ± 17.61	100.04 ± 15.45	102.07 ± 16.01
PANSS positive	22.22 ± 6.08	22.04 ± 6.42	21.21 ± 5.95
PANSS negative	27.27 ± 8.23	31.19 ± 5.94	34.14 ± 5.35
PANSS general	43.95 ± 8.94	46.81 ± 8.62	46.71 ± 8.43
BNSS total	40.29 ± 17.24	50.04 ± 10.70	57.00 ± 6.67
Anhedonia	8.98 ± 4.51	11.07 ± 3.29	13.07 ± 1.82
Lack of normal distress	2.76 ± 1.37	3.15 ± 1.41	3.79 ± 1.19
Asociality	6.32 ± 2.94	7.81 ± 1.80	8.36 ± 1.45
Avolition	6.49 ± 2.58	7.67 ± 2.06	8.50 ± 1.56
Blunted affect	10.29 ± 5.72	13.15 ± 4.37	14.93 ± 2.37
Alogia	5.46 ± 3.87	7.19 ± 3.37	8.36 ± 1.98
MAP	21.78 ± 8.85	26.56 ± 5.51	29.93 ± 3.79
EXP	15.76 ± 8.96	20.33 ± 6.87	23.29 ± 4.12

*Note:* SCZ, full schizophrenia group; SNS1, broad subgroup with severe negative symptoms; SNS2, restrictive subgroup with severe negative symptoms. Values are means ± *SD*.

#### Relationship between negative symptoms and biomarkers

**Figure 1.**
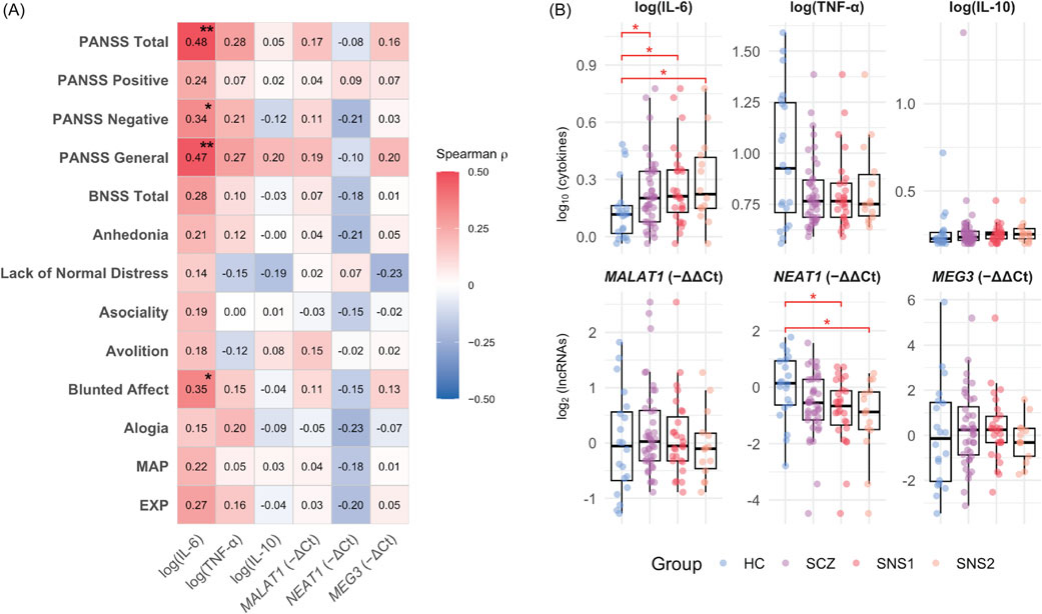
Cytokine–lncRNA associations with symptom dimensions and group differences. *Note:* A. Heatmap of Spearman correlations between biomarkers (IL-6, TNF-α, IL-10; lncRNAs *MALAT1*, *NEAT1*, *MEG3*) and symptom measures (PANSS Negative, PANSS Positive, PANSS General, BNSS). Colour scale: red = positive, blue = negative; *p* < 0.05 (*), *p* < 0.01 (**). Correlations were computed on log-transformed biomarker values. IL-6 shows visible positive associations with overall severity – especially PANSS Negative/General – whereas *NEAT1*, though not significant, trends negatively with negative-symptom measures. B. Box-and-jitter plots of log-transformed biomarker levels across groups (HC = healthy control group; SCZ = full schizophrenia group; SNS1 = broad subgroup with severe negative symptoms; SNS2 = restrictive subgroup with severe negative symptoms). Pairwise comparisons vs HC use Welch’s t test or Wilcoxon rank-sum as appropriate; asterisks indicate *p* < 0.05. Groups are displayed left-to-right (HC → SCZ → SNS1 → SNS2) to illustrate the direction of biomarker change across increasing negative-symptom severity.

IL-6 correlated positively with total PANSS scores (*ρ* = 0.48, *p* = 0.001), the PANSS Negative subscale (*ρ* = 0.34, *p* = 0.032), and BNSS Blunted Affect (*ρ* = 0.35, *p* = 0.026), with a trend for BNSS Total (*ρ* = 0.28, *p* = 0.072). Among non-negative domains, IL-6 was associated only with PANSS General Psychopathology (*ρ* = 0.47, *p* = 0.002). Group comparisons showed elevated IL-6 levels in SCZ (*M* = 1.70) versus HC (*M* = 1.38; GMR = 1.23, 95% CI [1.02, 1.50], *p* = 0.033) and a marginal *NEAT1* downregulation (FC = 0.65, 95% CI [0.42, 1.03], *p* = 0.064). Stratification by BNSS-defined severity amplified these effects: in SNS1, IL-6 was higher (GMR = 1.32, 95% CI [1.05, 1.64], *p* = 0.019) and *NEAT1* lower (FC = 0.56, 95% CI [0.34, 0.91], *p* = 0.020) than in HC, with no differences between non-SNS1 and HC. Similarly, in SNS2, IL-6 was elevated (GMR = 1.41, 95% CI [1.04, 1.90], *p* = 0.033) and *NEAT1* downregulated (FC = 0.46, 95% CI [0.24, 0.90], *p* = 0.025) versus HC, while non-SNS2 did not differ significantly. Detailed statistics are available in Supplements 2.1 and 2.2. Taken together, these unadjusted comparisons align with a severity-related pattern – higher IL-6 and lower *NEAT1* across HC → SCZ → BNSS-defined subgroups – although the group distributions overlap substantially, as shown in Figure 1.

#### Cytokine-lncRNA interrelationship and their association with the socio-demographic and clinical covariates

**Figure 2.**
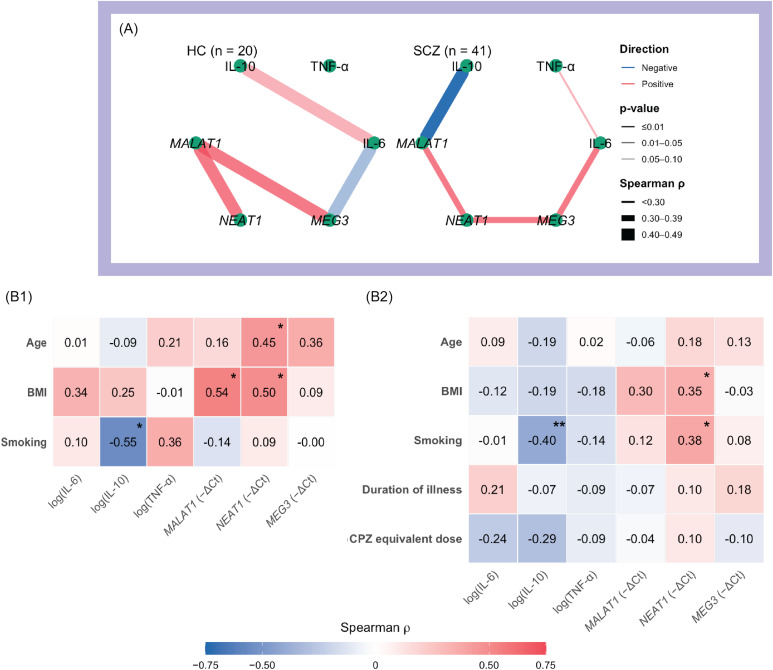
Cytokine–lncRNA interrelationships and links to socio-demographic/clinical covariates. *Note:* A. Network maps of Spearman correlations among biomarkers (cytokines: IL-6, TNF-α, IL-10; lncRNAs: *MALAT1*, *NEAT1*, *MEG3*) shown separately for healthy controls (HC) and the full schizophrenia group (SCZ). Edge colour indicates direction (red = positive; blue = negative); edge width is proportional to |ρ|; and edge opacity reflects significance (darker = smaller *p*). Biomarker values were log-transformed; sample size (*n*) per panel is indicated in the figure. B1–B2. Heatmaps of Spearman correlations between biomarkers and continuous covariates for HC (B1) and SCZ (B2). Colour scale: red = positive, blue = negative. Asterisks denote *p* < 0.05 (*) and *p* < 0.01 (**).

Among cytokines, only IL-6 showed marginal associations with IL-10 in HC (*ρ* = 0.44, *p* = 0.054) and with TNF-α in the SCZ group (*ρ* = 0.29, *p* = 0.067). By contrast, several moderate cytokines–lncRNA and lncRNA–lncRNA associations were observed. In HCs, *MALAT1* correlated positively with both *NEAT1* (*ρ* = 0.45, *p* = 0.046) and *MEG3* (*ρ* = 0.45, *p* = 0.044). In SCZ, IL-6 correlated positively with *MEG3* (*ρ* = 0.39, *p* = 0.013), IL-10 correlated negatively with *MALAT1* (*ρ* = –0.40, *p* = 0.009), and *NEAT1* correlated positively with both *MALAT1* (*ρ* = 0.38, *p* = 0.015) and *MEG3* (*ρ* = 0.33, *p* = 0.041). Among HCs, females had higher IL-6 levels than males (GMR = 1.40, 95% CI [1.01, 1.95], *p* = 0.044); IL-10 was inversely related to smoking (*ρ* = –0.55, *p* = 0.012); *MALAT1* was inversely related to BMI (*ρ* = –0.54, *p* = 0.015); and *NEAT1* correlated positively with age (*ρ* = 0.45, *p* = 0.048) and BMI (*ρ* = 0.50, *p* = 0.026). In the SCZ group, IL-6 was higher among single participants (GMR = 1.27, 95% CI [1.01–1.63], *p* = 0.036), while IL-10 levels were higher in individuals with university education (Kruskal–Wallis, *p* = 0.005) and in those with urban residence (GMR = 1.06, 95% CI [1.00, 1.15], *p* = 0.033); IL-10 was also negatively associated with smoking (*ρ* = –0.40, *p* < 0.001); *NEAT1* correlated positively with BMI (*ρ* = 0.35, *p* = 0.024) and smoking (*ρ* = 0.38, *p* = 0.013). Antipsychotic class, clozapine use, and CPZ-equivalent dose were not significantly related to any biomarker (all *p* ≥ 0.16). Network plots and correlation heatmaps summarising these associations are shown in Figure 2.

### Discriminative modelling of SCZ and BNSS subgroups using IL-6 and NEAT1

All logistic models satisfied key assumptions. Multicollinearity was minimal (maximum VIF = 2.04), with no evidence of complete or quasi-complete separation. Cook’s distances identified at most two moderately influential observations per model (maximum ≈ 0.7), none of which altered inferences. Calibration was acceptable across all contrasts (slopes ≈ 1.0; intercepts ≈ 0), and Hosmer–Lemeshow tests were non-significant (*p* ≥ 0.15). Linearity in the logit was supported for all predictors.

#### Healthy-control contrasts (covariate-adjusted binary models)

**Figure 3.**
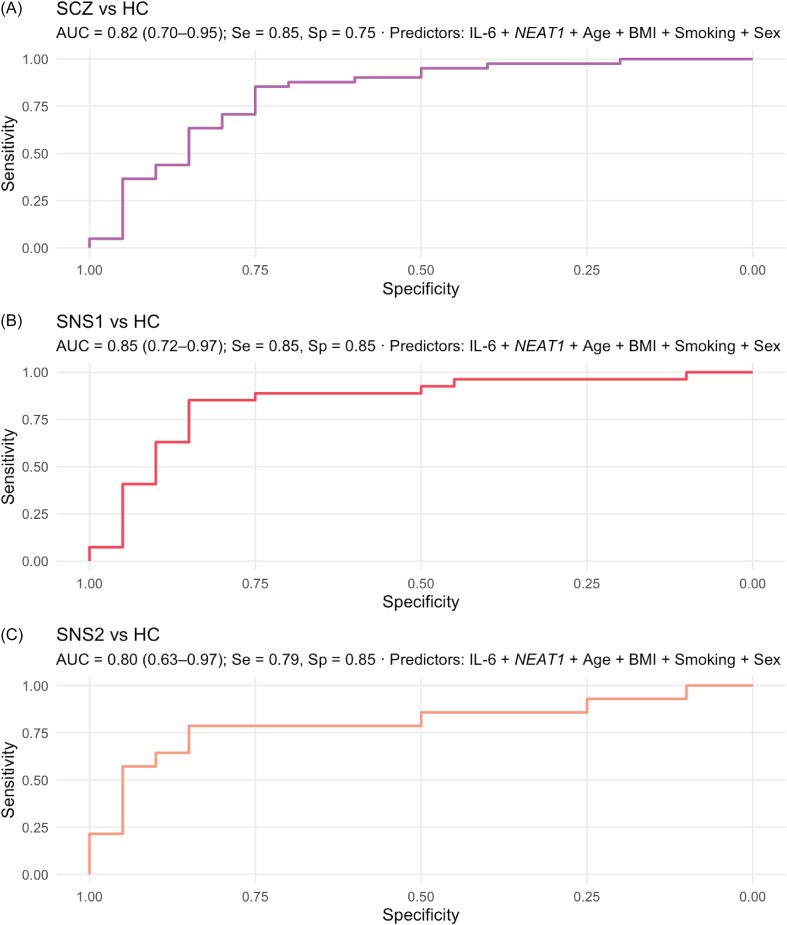
Receiver-operating characteristic (ROC) curves for a two-marker model. *Note:* ROC curves from covariate-adjusted linear logistic regression combining log(IL-6) and *NEAT1* (adjusted for age, sex, BMI, and smoking) to discriminate: A. SCZ vs HC, B. SNS1 vs HC, and C. SNS2 vs HC. Subpanels report AUC (DeLong 95% CI) and sensitivity/specificity at Youden’s J. Performance was highest for SNS1 (AUC = 0.85, 95% CI 0.72–0.97), with comparable accuracy for SCZ vs HC (AUC = 0.82, 95% CI 0.70–0.95) and SNS2 vs HC (AUC = 0.80, 95% CI 0.63–0.97). Abbreviations: HC = healthy control group; SCZ = full schizophrenia group; SNS1 = broad subgroup with severe negative symptoms; SNS2 = restrictive subgroup with severe negative symptoms.

Across all HC contrasts, dual-marker models demonstrated strong and well-calibrated discrimination (AUC = 0.80–0.85; Brier = 0.15–0.16). In SCZ versus HC (*n* = 61), model performance was good (AUC = 0.82, 95% CI [0.70–0.95]), with significant contributions from *NEAT1* (*p* = 0.001), IL-6 (*p* = 0.018), and smoking (*p* = 0.015); age, sex, and BMI were non-significant (*p* ≥ 0.12). An increase across one IQR in IL-6 was associated with 3.25-fold higher odds of SCZ versus HC (95% CI [1.08–9.78]), whereas an equivalent increase in *NEAT1* was associated with lower odds (OR_IQR = 0.17, 95% CI [0.05–0.64]). The SNS1 versus HC model (*n* = 47) achieved considerable discrimination (AUC = 0.85, 95% CI [0.72–0.97]), with both IL-6 (*p* = 0.013) and *NEAT1* (*p* = 0.002) significant, and smoking showing a trend (*p* = 0.063). For SNS2 versus HC (*n* = 34), discrimination was comparable (AUC = 0.80, 95% CI [0.63–0.97]), with *NEAT1* (*p* = 0.047) and IL-6 (*p* = 0.056) maintaining consistent directions. Smoking, sex, and BMI were non-significant (*p* > 0.40). Detailed results are presented in Table 4, and ROC curves for each model are shown in Figure 3.

**Table 4.** Covariate-adjusted logistic regression models for healthy-control contrasts (HC vs SCZ, SNS1, and SNS2) using IL-6 and *NEAT1* biomarkers

Group comparison	Term tested	Effect OR (95% CI)	Chi-sq	LR *p*	Wald *p*
SCZ vs HC (biomarkers + covariates)	IL-6 (IQR, log10)	3.25 (1.08–9.78)	5.61	0.018	0.036
	*NEAT1* (IQR, log2)	0.17 (0.05–0.64)	10.10	0.001	0.009
	Age (IQR)	2.79 (0.71–10.88)	2.43	0.119	0.140
	BMI (IQR)	1.33 (0.57–3.13)	0.47	0.493	0.506
	Smoking (Yes vs No)	5.21 (1.25–21.76)	5.91	0.015	0.024
	Sex (Male vs Female)	2.11 (0.50–8.93)	1.08	0.299	0.311
SNS1 vs HC (biomarkers + covariates)	IL-6 (IQR, log10)	3.93 (1.15–13.47)	6.22	0.013	0.029
	*NEAT1* (IQR, log2)	0.20 (0.06–0.71)	9.18	0.002	0.013
	Age (IQR)	2.71 (0.60–12.27)	1.85	0.174	0.195
	BMI (IQR)	0.84 (0.42–1.70)	0.22	0.638	0.635
	Smoking (Yes vs No)	4.22 (0.85–20.89)	3.47	0.063	0.077
	Sex (Male vs Female)	2.13 (0.43–10.64)	0.89	0.346	0.358
SNS2 vs HC (biomarkers + covariates)	IL-6 (IQR, log10)	3.83 (0.82–17.89)	3.64	0.056	0.088
	*NEAT1* (IQR, log2)	0.31 (0.09–1.12)	3.96	0.047	0.075
	Age (IQR)	3.09 (0.44–21.76)	1.41	0.235	0.258
	BMI (IQR)	0.62 (0.18–2.15)	0.59	0.441	0.455
	Smoking (Yes vs No)	1.95 (0.28–13.59)	0.45	0.501	0.502
	Sex (Male vs Female)	1.82 (0.23–14.23)	0.34	0.558	0.567

*Note:* HC, healthy control group; SCZ, full schizophrenia group; SNS1, broad subgroup with severe negative symptoms; SNS2, restrictive subgroup with severe negative symptoms; OR, odds ratio. IQR-OR denotes the odds ratio for increasing the predictor from its 25th to 75th percentile in the analysis dataset. LR test, likelihood-ratio drop-one test for each predictor (*df* = 1). Wald *p*, coefficient-level Wald test. References: Smoking, No; Sex, Female; baseline, HC.

#### Multinomial sensitivity analyses (SCZ subgroups and HC within unified models)

Multinomial logistic models including HC, non-SNS, and SNS subgroups confirmed consistent IL-6 and *NEAT1* effects across all contrasts, suggesting a graded biomarker pattern. In the SNS1/non-SNS1/HC model, likelihood-ratio tests indicated significant effects for *NEAT1* (*p* = 0.005), IL-6 (*p* = 0.021), and smoking (*p* = 0.043), while age, sex, and BMI were non-significant. Within the same model, higher IL-6 (OR_IQR = 4.41) and lower *NEAT1* (OR_IQR = 0.14) distinguished SNS1 most strongly from controls. Similarly, in the SNS2/non-SNS2/HC model, *NEAT1* (*p* = 0.003) and IL-6 (*p* = 0.030) remained significant overall, with consistent directions (IL-6 ↑, *NEAT1* ↓) and a modest effect of smoking (*p* = 0.050). IL-6 (OR_IQR = 4.98) and *NEAT1* (OR_IQR = 0.11) exhibited the steepest gradients compared to HCs. Full statistical results appear in Supplements 3.1 and 3.2.

## Discussion

This study developed and evaluated discriminative models using a peripheral cytokine–lncRNA panel to distinguish SCZ and BNSS-defined negative-symptom subgroups from HCs. Among the broader set of inflammatory (IL-6, TNF-α, IL-10) and nuclear lncRNA (*MALAT1, NEAT1, MEG3*) candidates, a dual-marker profile – characterised by elevated IL-6 and reduced *NEAT1* – showed the most consistent discrimination in models adjusted for age, sex, BMI, and smoking.

Negative symptoms were highly prevalent in our inpatient SCZ sample: approximately two-thirds met the broad BNSS-defined negative criteria (SNS1), and about one-third met the restrictive definition (SNS2). In a previous study, we demonstrated that BNSS, even under its restrictive severe-negative criteria, identifies more patients and measures more severe negative symptoms compared to PANSS, including among moderately psychotic inpatients (Moga *et al*., 2025). The ∼ 30% proportion under the restrictive definition aligns with the prevalence of the persistent negative symptoms construct in the literature (Buchanan, 2007). Across BNSS domains, lower BMI was associated with more severe negative symptoms, consistent with prior evidence of an inverse BMI–negative symptom association in chronic SCZ (Mezquida *et al*., 2018). We also observed an inverse association with mean CPZ-equivalent dose, which suggests that severe negative symptoms in this sample are unlikely to be secondary to higher antipsychotic exposure.

Across covariate-adjusted binary logistic models, discrimination was consistently considerable (AUC = 0.80–0.85) with good calibration. The broad severe negative-symptom subgroup (SNS1) achieved the best performance (AUC = 0.85), with balanced sensitivity and specificity (both 0.85). In contrast, the restrictive SNS2 definition showed slightly lower discrimination (AUC = 0.80), with the same specificity (0.85) but reduced sensitivity (0.79). Likelihood-ratio and Wald tests indicated significant contributions of IL-6 and *NEAT1* in the SCZ versus HC and SNS1 versus HC contrasts, with consistent directional patterns (elevated IL-6 and reduced *NEAT1*) across all models. Smoking contributed modestly, while age, sex, and BMI were generally non-influential after adjustment. Previous studies assessing cytokine-based discrimination – particularly IL-6 – between individuals with SCZ and HC have reported comparable performance, with AUC values ranging from 0.76 (Chase *et al*., 2016) to 0.96 (Shafiee-Kandjani *et al*., 2024) for IL-6 alone (at either the protein or transcript level) and exceeding 0.90 when combined with other cytokines (Liu *et al*., 2020). The present work extends these findings by, to our knowledge, being the first to evaluate discrimination based on negative-symptom stratification and to integrate IL-6 with a long noncoding RNA biomarker.

The complementary multinomial sensitivity analyses, which modelled HC, non-SNS, and SNS categories within a unified predictive framework, confirmed that the dual-marker combination of elevated IL-6 and reduced *NEAT1* maintained consistent effects across all contrasts. Complementing the pairwise tests, which showed significant differences primarily between SNS and HC, the multinomial models jointly evaluated all groups while adjusting for covariates, revealing that biomarker variation followed a graded, severity-dependent continuum rather than discrete diagnostic boundaries. The steepest odds ratios relative to HC were observed for the restrictive SNS2 and broad SNS1 subgroups for IL-6 (OR_IQR = 4.98 and 4.41, respectively) and for *NEAT1* reductions (OR_IQR = 0.11 and 0.14), with intermediate shifts in non-SNS participants. Together, these findings suggest that the IL-6/*NEAT1* combination reflects a continuous, severity-related gradient of immune–molecular alteration across the SCZ spectrum and that the multinomial modelling approach strengthens the evidence for its discriminative consistency across subgroup boundaries.

Beyond statistical discrimination, IL-6 levels tracked symptom burden – correlating with PANSS Total, Negative, and General scores, as well as BNSS Blunted Affect – and were elevated in SCZ compared to HC, with the largest increases observed among BNSS-defined negative subgroups. This pattern aligns with prior work describing IL-6 as a state-like inflammatory load in subsets of SCZ (Reale *et al*., 2021). Previous studies have similarly localised elevated IL-6 to specific ‘inflammatory’ SCZ subgroups (Boerrigter *et al*., 2017; Zhu *et al*., 2022; Alexandros Lalousis *et al*., 2023), characterised mainly by severe negative symptoms (Garcia-Rizo *et al*., 2012; Goldsmith *et al*., 2018; Wang *et al*., 2022; Cyran *et al*., 2023). Mechanistically, IL-6 has been linked to peripheral and central immune activation (Zhou *et al*., 2021), BBB disruption (Lv & Luo, 2025), dopamine downregulation (Goldsmith & Rapaport, 2020; Varela *et al*., 2023), and alterations in the tryptophan–kynurenine pathway (Schwieler *et al*., 2014; Plitman *et al*., 2017), providing plausible routes from systemic inflammation to motivational and expressive deficits. These mechanisms parallel the ‘sickness behavior syndrome’ from cytokine-induced depression models, in which lipopolysaccharide or IL-1 administration induces social withdrawal, reduced activity, and lethargy (Bluthé et al., 2000; Dantzer *et al*., 2008) – features overlapping with negative symptoms. This translational bridge suggests that the cytokine–depression model offers a biologically relevant framework for understanding negative symptoms in SCZ (Gangadin *et al*., 2024). Nonetheless, the cross-sectional design of the current study precludes causal inference.

*NEAT1* was downregulated within BNSS-defined negative subgroups and contributed to the discrimination between SCZ and HC in adjusted models. In contrast to our findings, *NEAT1* upregulation has been reported in autoimmune and neurodegenerative diseases (Zhang *et al*., 2019; Plewka & Raczynska, 2022), where it is linked to pro-inflammatory signalling. Conversely, reduction or knockdown of the long *NEAT1*_2 isoform causes paraspeckle disintegration and altered nuclear architecture (Yamazaki & Hirose, 2015; Almalki, 2024). Katsel et al. demonstrated that *NEAT1* reduction in SCZ is linked to dysregulation of the oligodendrocyte lineage and changes in myelin-related genes in both murine models and post-mortem human frontal cortex, emphasising *NEAT1*’s role in oligodendrocytes and myelin biology (Katsel *et al*., 2019). Similarly, *Neat1* ^-^/^-^ knockout mice have been shown to exhibit heightened stress reactivity and impaired social behaviour (Kukharsky *et al*., 2020), suggesting that *NEAT1* insufficiency may represent a trait-like vulnerability that amplifies stress-related signalling and contributes to social–motivational deficits. We interpret *NEAT1* as a trait-leaning, hypothesis-generating marker that complements IL-6 rather than a definitive subtype determinant.

These results have intertwined clinical and research implications. A concise whole-blood dual-marker panel – defined by higher IL-6 and lower *NEAT1* – retained significant discriminative value after adjustment for age, sex, BMI, and smoking, with the strongest performance for SNS1 versus HC and consistent directions for SNS2. Clinically, the panel could complement the BNSS by providing a peripheral correlate of negative-symptom burden, supporting biologically informed subgroup stratification and longitudinal monitoring. From a research perspective, it offers a practical platform for prediction-based trial design targeting negative symptoms and for assessing target engagement in mechanistic studies. In this dataset, the restrictive BNSS subgroup (SNS2) did not achieve higher accuracy than SNS1, suggesting a severity-related continuum rather than a discrete category. Given the modest, single-site design, these findings should be regarded as hypothesis-generating. Future studies should prioritise external validation in longitudinal BNSS-defined or related negative-symptom cohorts to confirm the robustness and clinical utility of this approach.

Several limitations merit consideration when interpreting the findings of this study: 1. The SCZ sample was modest and heterogeneous: all participants were hospitalised and under chronic antipsychotic treatment, predominantly clozapine, which may limit generalisability beyond inpatient settings. Nonetheless, in Romania’s hospital-centric system, the most severely burdened SCZ cases, including those with pronounced negative symptoms, are primarily recruited in hospitals. 2. The HC sample was relatively small, and the exclusion of individuals with a family history of psychosis, while reducing biological heterogeneity, may overestimate case–control differences. Future work should include population-based controls (with and without familial risk) or conduct sensitivity analyses. 3. The cross-sectional design precludes causal inference both between symptom severity and biomarkers and between inflammatory and transcriptional compartments; longitudinal data are needed to test directionality. 4. Negative symptoms were defined using BNSS-derived criteria (SNS1/SNS2) rather than longitudinal constructs such as persistent negative symptoms or deficit SCZ, which are typically assessed with the Schedule for the Deficit Syndrome (SDS). This difference may limit direct comparability with SDS-based literature. 5. Finally, mechanistic resolution was limited and beyond the scope of this study: we did not dissect specific signalling pathways underlying *NEAT1* downregulation or IL-6 elevation, and whole-blood means obscure cell-type and isoform specificity. Future work should integrate isoform-aware assays, cell sorting or single-cell profiling, and longitudinal validation.

Three key conclusions emerge from this study: 1. Discriminative performance: In covariate-adjusted linear models, confirmed by multinomial analyses, the dual-marker model of elevated IL-6 and reduced *NEAT1* showed significant discriminative value for SCZ diagnosis and BNSS-defined negative subgroups versus controls, with strong calibration and consistent directional effects. 2. Phenotypic gradient: The broad BNSS subgroup (SNS1) showed the strongest discrimination versus the controls, while SNS2 exhibited the steepest odds ratios in multinomial models – suggesting a severity-dependent gradient across the SCZ spectrum. 3. Conceptual framework: These findings reinforce a biomarker-based classification framework integrating inflammatory (IL-6) and nuclear-regulatory (*NEAT1*) pathways, establishing a translational basis for longitudinal validation and for evaluating the clinical, therapeutic, and mechanistic relevance of combined cytokine–lncRNA biomarkers.

## Supporting information

Moga et al. supplementary material 1 Moga et al. supplementary material

Moga et al. supplementary material 2 Moga et al. supplementary material

Moga et al. supplementary material 3 Moga et al. supplementary material

## Supplementary material

The supplementary material for this article can be found at https://doi.org/10.1017/neu.2026.10055.
