# Supplementary figures and images for: A dual-marker peripheral signature of IL-6 elevation and NEAT1 reduction in negative-symptom schizophrenia: a cross-sectional study
Source: Acta Neuropsychiatr. 2026 Jan 23;38:e13. doi: 10.1017/neu.2026.10055 (PMC13130349; doi:10.1017/neu.2026.10055)

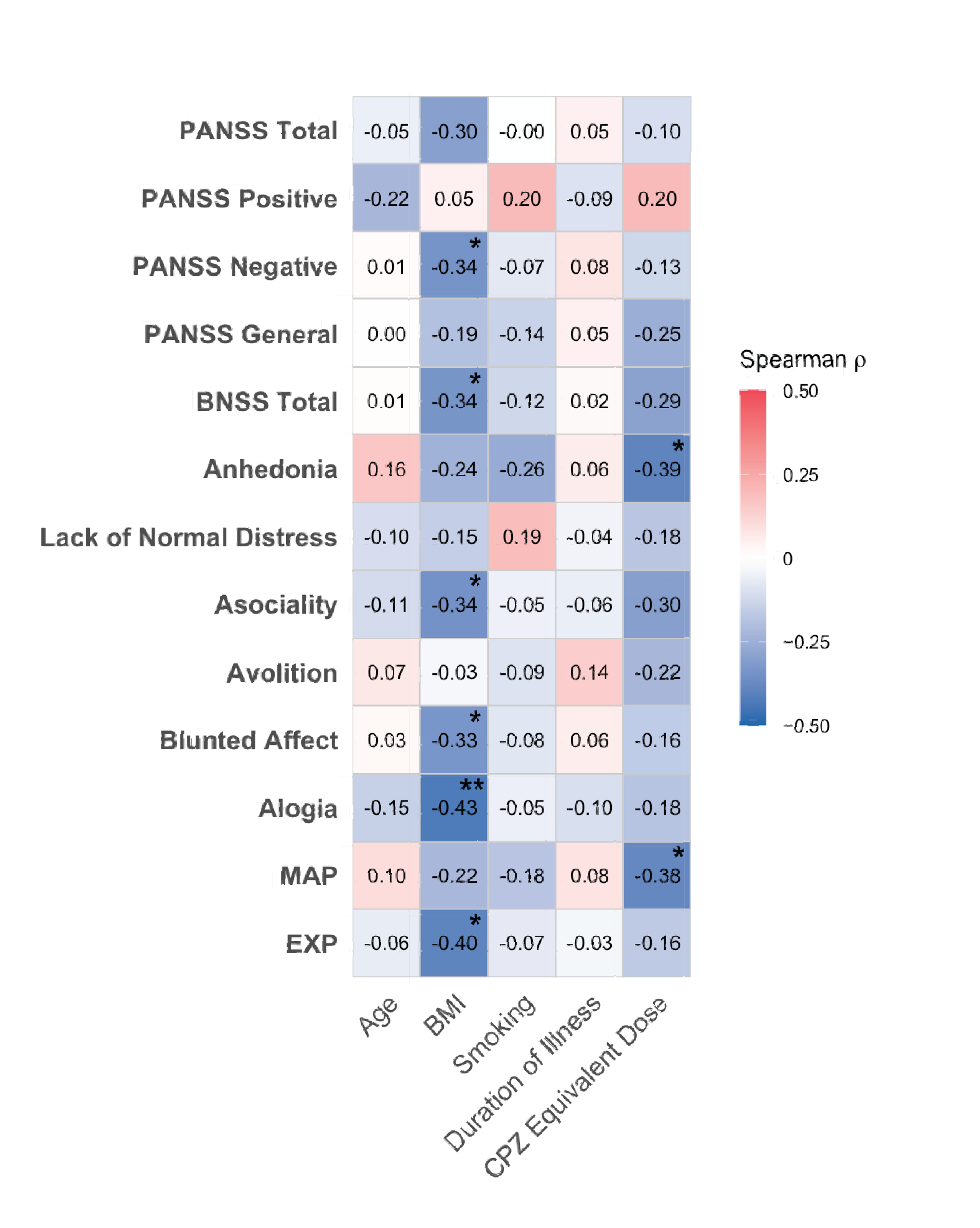

Supplement: Moga et al. supplementary material 1 — Moga et al. supplementary material [file S0924270826100556sup001.tiff]
