# Supplementary material for: A dual-marker peripheral signature of IL-6 elevation and NEAT1 reduction in negative-symptom schizophrenia: a cross-sectional study
Source: Acta Neuropsychiatr. 2026 Jan 23;38:e13. doi: 10.1017/neu.2026.10055 (PMC13130349; doi:10.1017/neu.2026.10055)
Supplement: Moga et al. supplementary material 2 — Moga et al. supplementary material [file S0924270826100556sup002.docx]

| **Supplement 2.1. Cytokine and lncRNA Levels across SNS1 Subgroups and Healthy Controls** | | | | | | |
| --- | --- | --- | --- | --- | --- | --- |
| **Biomarker Comparison** | **Means** | | | **Group Differences** | | |
| **Cytokine (pg/ml)** | HC | non-SNS1 | SNS1 | non-SNS1 vs HC | SNS1 vs non-SNS1 | SNS1 vs HC |
| IL-6 | 1.38 | 1.47 | 1.83 | *W* = 110, *p* = 0.302 | *W* = 141, *p* = 0.191 | *W* = 161, *p* = 0.019 |
| TNF-α | 9.54 | 6.45 | 6.41 | *W* = 171.5, *p* = 0.278 | *W* = 194, *p* = 0.902 | *W* = 333, *p* = 0.179 |
| IL-10 | 1.84 | 2.13 | 1.80 | *W* = 123, *p* = 0.562 | *W* = 178, *p* = 0.772 | *W* = 226.5, *p* = 0.353 |
| **lncRNA (FC ΔΔ Ct)** |  | | | | | |
| *MALAT1* | 1.00 | 1.31 | 1.09 | *W* = 106, *p* = 0.241 | *W* = 212, *p* = 0.536 | *W* = 239, *p* = 0.512 |
| *NEAT1* | 1.00 | 0.89 | 0.56 | *t* = 0.44, *p* = 0.663 | *W* = 246, *p* = 0.12 | *t* = 2.41, *p* = 0.020 |
| *MEG3* | 1.00 | 1.15 | 1.23 | *t* = -0.28, *p* = 0.782 | *t* = -0.15, *p* = 0.884 | *t* = -0.49, *p* = 0.629 |
| **Note.** HC = healthy control group; non-SNS1 = schizophrenia subgroup not meeting SNS1 criteria; SNS1 = broad subgroup with severe negative symptoms. Values are geometric means (back-transformed): cytokines from log₁₀-transformed data; lncRNAs from log₂-transformed data. Statistical tests were conducted on log-transformed values. *t* = Welch’s t-test statistic (used for normally distributed variables, per Shapiro–Wilk test); *W* = Wilcoxon rank-sum statistic (used for non-normal distributions). | | | | | | |

| **Supplement 2.2. Cytokine and lncRNA Levels across SNS2 Subgroups and Healthy Controls** | | | | | | | |
| --- | --- | --- | --- | --- | --- | --- | --- |
| **Biomarker Comparison** | **Means** | | | **Group Differences** | | |  |
| **Cytokine (pg/ml)** | HC | non-SNS2 | SNS2 | non-SNS2 vs HC | SNS2 vs non-SNS2 | SNS2 vs HC |  |
| IL-6 | 1.38 | 1.58 | 1.96 | *W* = 192.5, *p* = 0.0975 | *W* = 142, *p* = 0.201 | *W* = 78.5, *p* = 0.033 |  |
| TNF-α | 9.54 | 6.25 | 6.77 | *W* = 335.5, *p* = 0.162 | *W* = 183.5, *p* = 0.891 | *W* = 169, *p* = 0.319 |  |
| IL-10 | 1.84 | 1.94 | 1.83 | *W* = 238.5, *p* = 0.503 | *W* = 165, *p* = 0.516 | *W* = 111, *p* = 0.316 |  |
| **lncRNA (FC ΔΔ Ct)** |  | | | | | | |
| *MALAT1* | 1.00 | 1.28 | 0.97 | *W* = 207, *p* = 0.179 | *W* = 244, *p* = 0.134 | *W* = 138, *p* = 0.958 |  |
| *NEAT1* | 1.00 | 0.78 | 0.46 | *t* = 1.12, *p* = 0.272 | *W* = 246, *p* = 0.12 | *t* = 2.39, *p* = 0.025 |  |
| *MEG3* | 1.00 | 1.41 | 0.86 | *t* = -0.78, *p* = 0.439 | *t* = 1.58, *p* = 0.123 | *t* = 0.38, *p* = 0.709 |  |
| **Note.** HC = healthy control group; non-SNS2 = schizophrenia subgroup not meeting SNS2 criteria; SNS2 = restrictive subgroup with severe negative symptoms. Values are geometric means (back-transformed): cytokines from log₁₀-transformed data; lncRNAs from log₂-transformed data. Statistical tests were conducted on log-transformed values. *t* = Welch’s t-test statistic (used for normally distributed variables, per Shapiro–Wilk test); *W* = Wilcoxon rank-sum statistic (used for non-normal distributions). | | | | | | | |
