# Supplementary material for: A dual-marker peripheral signature of IL-6 elevation and NEAT1 reduction in negative-symptom schizophrenia: a cross-sectional study
Source: Acta Neuropsychiatr. 2026 Jan 23;38:e13. doi: 10.1017/neu.2026.10055 (PMC13130349; doi:10.1017/neu.2026.10055)
Supplement: Moga et al. supplementary material 3 — Moga et al. supplementary material [file S0924270826100556sup003.docx]

| **Supplement 3.1. Covariate-adjusted multinomial logistic regression comparing HC, non-SNS1, and SNS1 schizophrenia subgroups using IL-6 and *NEAT1* biomarkers.** | | | | | |
| --- | --- | --- | --- | --- | --- |
| **Group comparison** | **Term tested** | **Effect OR (95% CI)** | **Chi-sq** | **LR *p*** | **Wald *p*** |
| SNS1 vs HC (multinomial; biomarkers + covariates) | IL-6 (IQR, log10) | 4.41 (1.28–15.18) | 7.76 | 0.021 | 0.019 |
|  | *NEAT1* (IQR, log2) | 0.14 (0.04–0.59) | 10.73 | 0.005 | 0.007 |
|  | Age (IQR) | 2.92 (0.69–12.33) | 2.40 | 0.302 | 0.145 |
|  | BMI (IQR) | 0.97 (0.35–2.71) | 2.97 | 0.226 | 0.956 |
|  | Smoking (Yes vs No) | 4.27 (0.89–20.52) | 6.28 | 0.043 | 0.070 |
|  | Sex (Male vs Female) | 2.43 (0.50–11.81) | 1.33 | 0.514 | 0.270 |
| non-SNS1 vs HC (multinomial; biomarkers + covariates) | IL-6 (IQR, log10) | 1.66 (0.40–6.96) | 7.76 | 0.021 | 0.487 |
|  | *NEAT1* (IQR, log2) | 0.30 (0.07–1.34) | 10.73 | 0.005 | 0.116 |
|  | Age (IQR) | 2.37 (0.47–12.00) | 2.40 | 0.302 | 0.298 |
|  | BMI (IQR) | 1.90 (0.73–4.96) | 2.97 | 0.226 | 0.190 |
|  | Smoking (Yes vs No) | 6.79 (1.26–36.59) | 6.28 | 0.043 | 0.026 |
|  | Sex (Male vs Female) | 1.41 (0.23–8.66) | 1.33 | 0.514 | 0.709 |
| **Note.** HC = healthy control group; non-SNS1 = schizophrenia subgroup not meeting SNS1 criteria; SNS1 = broad subgroup with severe negative symptoms; OR = odds ratio. IQR-OR denotes the odds ratio for increasing the predictor from its 25th to 75th percentile in the analysis dataset. LR test = type-II likelihood-ratio test for each predictor across both logits (df = 2; outcomes = SNS1, non-SNS1, HC with HC as baseline). Wald *p* corresponds to the row’s specific logit (e.g., SNS vs HC or non-SNS vs HC). References: Smoking = No; Sex = Female. | | | | | |

| **Supplement 3.2. Covariate-adjusted multinomial logistic regression comparing HC, non-SNS2, and SNS2 schizophrenia subgroups using IL-6 and *NEAT1* biomarkers.** | | | | | |
| --- | --- | --- | --- | --- | --- |
| **Group comparison** | **Term tested** | **Effect OR (95% CI)** | **Chi-sq** | **LR *p*** | **Wald *p*** |
| SNS2 vs HC (multinomial; biomarkers + covariates) | IL-6 (IQR, log10) | 4.98 (1.31–18.92) | 6.99 | 0.030 | 0.018 |
|  | *NEAT1* (IQR, log2) | 0.11 (0.02–0.50) | 11.60 | 0.003 | 0.004 |
|  | Age (IQR) | 4.16 (0.82–21.08) | 3.27 | 0.195 | 0.085 |
|  | BMI (IQR) | 0.91 (0.25–3.24) | 1.37 | 0.504 | 0.881 |
|  | Smoking (Yes vs No) | 4.14 (0.65–26.53) | 5.98 | 0.050 | 0.133 |
|  | Sex (Male vs Female) | 2.36 (0.38–14.75) | 1.01 | 0.603 | 0.358 |
| non-SNS2 vs HC (multinomial; biomarkers + covariates) | IL-6 (IQR, log10) | 2.64 (0.83–8.40) | 6.99 | 0.030 | 0.100 |
|  | *NEAT1* (IQR, log2) | 0.22 (0.06–0.85) | 11.60 | 0.003 | 0.028 |
|  | Age (IQR) | 2.33 (0.57–9.61) | 3.27 | 0.195 | 0.242 |
|  | BMI (IQR) | 1.48 (0.61–3.54) | 1.37 | 0.504 | 0.384 |
|  | Smoking (Yes vs No) | 5.48 (1.27–23.72) | 5.98 | 0.050 | 0.023 |
|  | Sex (Male vs Female) | 1.90 (0.42–8.57) | 1.01 | 0.603 | 0.406 |
| **Note.** HC = healthy control group; non-SNS2 = schizophrenia subgroup not meeting SNS2 criteria; SNS2 = restrictive subgroup with severe negative symptoms; OR = odds ratio. IQR-OR denotes the odds ratio for increasing the predictor from its 25th to 75th percentile in the analysis dataset. LR test = type-II likelihood-ratio test for each predictor across both logits (df = 2; outcomes = SNS2, non-SNS2, HC with HC as baseline). Wald *p* corresponds to the row’s specific logit (e.g., SNS vs HC or non-SNS vs HC). References: Smoking = No; Sex = Female. | | | | | |
